# Cystic Fibrosis Gene Therapy in the UK and Elsewhere

**DOI:** 10.1089/hum.2015.027

**Published:** 2015-04-01

**Authors:** Uta Griesenbach, Kamila M. Pytel, Eric W.F.W. Alton

**Affiliations:** Department of Gene Therapy and the U.K. Cystic Fibrosis Gene Therapy Consortium, Imperial College, London SW3 6LR, United Kingdom.

## Abstract

The cystic fibrosis transmembrane conductance regulator (*CFTR*) gene was identified in 1989. This opened the door for the development of cystic fibrosis (CF) gene therapy, which has been actively pursued for the last 20 years. Although 26 clinical trials involving approximately 450 patients have been carried out, the vast majority of these trials were short and included small numbers of patients; they were not designed to assess clinical benefit, but to establish safety and proof-of-concept for gene transfer using molecular end points such as the detection of recombinant mRNA or correction of the ion transport defect. The only currently published trial designed and powered to assess clinical efficacy (defined as improvement in lung function) administered AAV2-CFTR to the lungs of patients with CF. The U.K. Cystic Fibrosis Gene Therapy Consortium completed, in the autumn of 2014, the first nonviral gene therapy trial designed to answer whether repeated nonviral gene transfer (12 doses over 12 months) can lead to clinical benefit. The demonstration that the molecular defect in CFTR can be corrected with small-molecule drugs, and the success of gene therapy in other monogenic diseases, is boosting interest in CF gene therapy. Developments are discussed here.

## Cystic Fibrosis: An Introduction to the Disease

Cystic fibrosis (CF) is an autosomal recessive disease caused by mutations in the cystic fibrosis transmembrane conductance regulator (*CFTR*) gene. The *CFTR* gene encodes a protein that has several functions including cAMP-dependent chloride and bicarbonate secretion and regulation of epithelial sodium channels (ENaCs). CFTR is, therefore, important in the regulation of ion and fluid homeostasis across epithelial barriers.^1^

The *CFTR* gene is expressed in the epithelium of many organs including the lungs, kidney, the gastrointestinal and reproductive tracts, liver, and pancreas leading to multiorgan disease.^2^ In the developed world progressive pulmonary disease causes most morbidity and mortality. The respiratory disease is characterized by persistent cycles of lung infection and inflammation leading to mucous obstruction of the airways. Although the role of CFTR in transepithelial ion transport and host defense is widely accepted, there is ongoing debate about some of the key processes. The “low volume” hypothesis,^3^ which postulates that decreased transepithelial chloride transport, due to mutated CFTR, and increased transepithelial sodium absorption, due to lack of CFTR-dependent inhibition of ENaCs, lead to increased water absorption into the tissue and, therefore, decreased airway surface liquid and reduced mucociliary clearance has long been the leading hypothesis. This hypothesis is consistent with altered potential difference measurements in the nose and upper airways of patients with CF.^4,5^ Studies in the CF knockout pig confirmed the lack of chloride transport and sodium hyperabsorption in nasal epithelium,^6^ but highlighted that sodium hyperabsorption and depletion of airway surface liquid were not present in CF pig lower airways,^7^ thereby somewhat questioning the validity of the “low volume” hypothesis. However, consistent with patients with CF, the CF pig also had reduced CFTR-dependent bicarbonate secretion in the airways,^7^ which in the pig leads to reduced airway surface pH causing impairment of innate bacterial defense mechanisms.^8^ The role of CFTR expression in inflammatory cells such as neutrophils, macrophages, and more recently T cells, has been widely, and so far, inconclusively debated,^9–13^ but studies overall appear to suggest a potential defect in adaptive immune responses in patients with CF, which may explain the exaggerated pulmonary inflammatory responses that have been generally observed, an area that requires further studies.

CF is the most common genetic disease in the white population. Approximately 80,000 people have been diagnosed with CF in the United States and Europe^14^ with 10,000 patients with CF living in the United Kingdom today, of which more than 57% are adults.^15^ However, increasing numbers of patients with CF are being identified in other large populations including China and India. CF was first defined as a disease in 1938, but the *CFTR* gene was not identified until 1989^16–18^; a landmark that opened the door for the development of CF gene therapy. To date more than 1990 mutations have been identified within the *CFTR* gene,^19^ but not all can be conclusively categorized as disease-causing. On the basis of the resulting cellular phenotype the mutations can be classified into six classes^2^ (Table 1). Classes I–III tend to abolish CFTR expression and/or function (severe mutations) whereas classes IV–VI produce CFTR variants with residual expression and/or function (mild mutations).^2^ Although the genotype–phenotype correlation is strong for pancreatic disease (severe mutations lead to pancreatic insufficiency and patients require enzyme supplements to digest food, whereas patients with mild mutations remain pancreatically sufficient), in the lung the environment, socioeconomic factors, and other modifier genes significantly contribute to disease severity. By far the most common mutation worldwide is a deletion of phenylalanine (Phe508del, previously called ΔF508). In the United Kingdom 90% of patients with CF are homozygous or compound heterozygous for Phe508del, although the absolute frequency varies among different populations. Several therapies including, among others, inhaled antibiotics, macrolides, and novel mucolytics such as dornase alfa (a recombinant DNase) and hypertonic saline, have progressed from clinical trials into mainstream treatment and led to a steady increase in median predicted survival, which is currently 37 years in the United Kingdom.^15^ The introduction of clinical trial networks in the United States^20^ and Europe^21^ has significantly contributed to the rapid progression from bench to bedside.

**Table 1.** *CFTR* Gene Mutation Classes

*Class*	*Effect on protein*	*Key example of disease-causing mutations*
I	Defective protein production	G542X
II	Defective protein processing	F508del
III	Defective protein regulation	G551D
IV	Defective protein conductance	R117H
V	Reduced protein synthesis	A455E
VI	Reduced protein surface retention	c.120del23

*CFTR*, cystic fibrosis transmembrane conductance regulator gene. Of note: a large number of known *CFTR* mutations are currently unclassified with respect to mutation class.

## Gene Therapy to Treat CF Lung Disease

As mentioned previously, the cloning of the *CFTR* gene was a landmark for the development of CF gene therapy. The vast majority of efforts over the last 20 years have focused on developing gene therapy for CF lung disease, largely due to the urgent need for more effective treatments and the noninvasive accessibility of the lung.

Identification of the now-licensed CFTR potentiator Kalydeco (also known as ivacaftor or VX-770) has been a success story for high-throughput small-molecule drug development. Kalydeco potentiates CFTR protein function in patients with class III gating mutations.^22,23^ However, it is important to note that only ∼4% of patients with CF carry mutations that respond to Kalydeco. The development of small-molecule drugs that improve CFTR processing (*correctors*) in patients with Phe508del mutations, which would be beneficial for the vast majority of patients with CF, has so far proven more difficult (reviewed in Bell et al.^24^). However, research into CFTR correctors and, more importantly the potentiator Kalydeco, has shown that the molecular defect of CFTR is drug targetable and that amelioration of CFTR function translates into improvements in disease phenotype. These findings have renewed interest and investment into other therapies aimed at targeting the molecular defect, including gene therapy.

Gene therapy for CF has a number of important features compared with other experimental and established drugs:

1. In contrast to the mutation class–specific treatments described previously, gene therapy is mutation class independent and will likely be suitable for all patients.
2. In contrast to many other established drugs, gene therapy will target the disease at its origin, the molecular defect. This offers the potential for lung disease prevention, if treatments are initiated early.
3. As described previously, disease pathophysiology is widely debated. Lung gene therapy will not require disease pathology to be completely illuminated. However, if CFTR expression in inflammatory cells turns out to be an important factor for host defense, additional gene therapy–based strategies such as transduction of bone marrow–derived cells may be important.

## Challenges for CF Gene Therapy

The development of pulmonary gene therapy has been slower than initially predicted and hoped for. The lung is a complex and difficult target organ because of the presence of potent intra- and extracellular barriers that have evolved to protect the lungs from foreign bodies including bacteria, viruses, and gene transfer agents. Extracellular mucus and CF sputum, cilia beating, and the nuclear membrane all affect the efficiency of gene transfer.^25^ In addition, a number of other questions are currently unresolved.

### What level of CFTR gene expression is required to achieve clinical benefit in the treated patient?

A body of evidence suggests that even modest amounts of CFTR expression may suffice to improve lung disease.

First, data from early *in vitro* studies suggest that low-level correction of 6–10% of *CFTR* gene expression in airway epithelium can restore chloride transport to non-CF levels.^26^ However, later studies indicated that higher numbers of CFTR-expressing cells (>25%) are necessary to allow efficient mucous transport of CFTR.^27^ More recently, adenovirus-mediated *CFTR* gene transfer into 20–30% of sinus epithelial cells extracted from CF knockout pigs corrected chloride transport to 50% of non-CF levels, and even low levels of gene transfer (∼7%) produced detectable levels of correction (∼6% of non-CF).^28^

Second, individuals with CF with certain “mild” mutations that retain as little as 10% of normal CFTR expression per cell generally do not suffer from lung disease, although other organs such as the vas deferens may be affected.^29^

However, it is currently unclear whether low-level expression in many cells (as in patients with 10% of residual CFTR expression) or a low number of cells expressing high levels of CFTR (as in the cell-mixing experiments) will be required to achieve clinical benefit after gene therapy. This question is further complicated by the fact that molecular assays to quantify mRNA and protein expression do not work particularly well on bronchial brushings and biopsies obtained from the lungs of patients with CF after gene transfer,^30,31^ due to (1) the small amount of sample that can be obtained, (2) the region that bronchial brushings and biopsies can be obtained from (comparatively large third- to fourth-generation airways, rather than smaller airways in which disease first manifests), (3) the poor specificity of anti-CFTR-specific antibodies, and (4) the relative sensitivity of the assays.

In addition, it is unclear which of the features related to CF pathophysiology are most important. None of the gene therapy trials reported so far has shown evidence for correction of the sodium transport defect, and *in vitro* cell-mixing experiments implied that the correction of sodium hyperabsorption requires high numbers (close to 100%) of non-CF cells.^32,33^ We are currently not aware of studies that have addressed how much CFTR expression in how many cells may be required to correct the bicarbonate transport defect and associated alterations in mucus unfolding^34^ and airway surface liquid pH.^8^

**FIG. 1.**
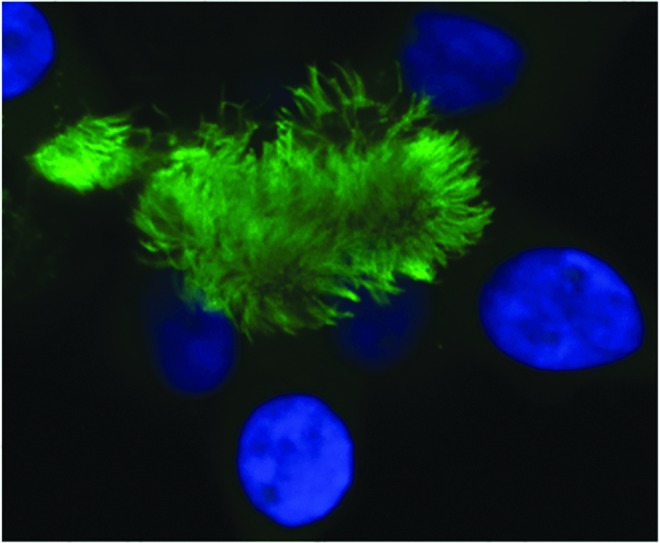
Ciliated human airway epithelial cells: target cells for CF gene therapy. The dense ciliary border on airway epithelial cells is a significant extracellular barrier to CF gene therapy. Cilia are stained with an anti-tubulin-β antibody (green). Nuclei are stained with DAPI (blue). Picture courtesy of M. Wasowicz (Department of Gene Therapy, Imperial College, London). Color images available online at www.liebertpub.com/hum

Another question that remains unresolved concerns which cells in the lung are the most appropriate target for gene transfer. CFTR is expressed in various lung regions and cell types,^35–39^ and in our view airway epithelial cells (Fig. 1) are currently the most likely target cell for gene replacement.

Twenty-six clinical trials involving approximately 450 patients have been carried out.^40^ A phrase similar to “CF gene therapy has failed to demonstrate clinical benefit,” which is frequently stated in the literature, requires some clarification. The vast majority of the CF gene therapy trials were short and included small numbers of patients and were not designed to assess clinical benefit, but to establish safety and proof-of-concept for gene transfer using molecular end points such as the detection of recombinant mRNA or correction of the ion transport defect. Early trials focused on delivery of gene transfer agents to the nasal epithelium, used as a surrogate for lung delivery because of their similarity in cell composition and being potentially safer for first-in-human studies. Importantly, these studies clarified the strength and weaknesses of viral and nonviral gene transfer for chronic diseases such as CF and highlighted that preclinical models do not well predict clinical trial outcomes. Landmark preclinical and clinical studies are described below.

## Viral Vector–Mediated Gene Transfer

### Early studies: Adenoviral vectors for CF gene therapy

Adenoviruses are nonenveloped, double-stranded DNA viruses composed of a complex icosahedral capsid.^41,42^ The knob domain of the fiber binds to the coxsackie and adenovirus receptor (CAR) on the cell surface and facilitates virus entry,^41,43^ although integrin receptors such as α_v_β_3_ and α_v_β_5_ and the major histocompatibility complex (MHC) class I may also play a role in virion–cell interaction.^43^ After entry the virus remains in an episomal state within the nucleus.

Because of a natural tropism for the lung and a large packaging capacity, adenoviral vectors were the first to be assessed for CF gene therapy. In first-generation vectors the E1 region was deleted to prevent virus replication. More advanced versions, so-called helper-dependent adenoviral (HDAd) vectors, were depleted of all viral coding sequences.^40,44^ The first, albeit not placebo-controlled, CF gene therapy trial was reported by Zabner and colleagues in 1993, only 4 years after cloning the *CFTR* gene. A serotype 2 adenoviral (Ad2) vector carrying the human *CFTR* cDNA was administrated to the nasal epithelium of three patients with CF. Although patient numbers were low, this study provided the first proof-of-concept for correction of the chloride transport defect and stimulated further interest in the development of adenovirus-based clinical trials.^45^ Since 1994, nine further adenoviral vector trials were conducted.^46–54^ Combined, these studies showed that adenoviral vectors were not suitable for CF gene therapy because of (1) inefficient and transient gene transfer and (2) induction of immune responses that prevented efficacy on repeat administration (as reviewed in Griesenbach and Alton^40^).

Numerous preclinical studies have been conducted to increase gene transfer and reduce induction of immune responses. The realization that the CAR receptors are located on the basolateral rather than the apical membrane of airway epithelial cells led to assessment of tight junction openers such as sodium caprate^55^ and lysophosphatidylcholines,^56^ which showed moderate success. In addition, various immunosuppressive strategies were assessed, but the suitability of tight junction openers and immunosuppression in the context of bacteria-infected CF lungs was never addressed systematically. The more recent development of HDAd vectors goes somewhat toward reducing inflammatory and immune responses,^57–59^ but progress has not been sufficient to warrant assessment of HDAd vectors in CF, a chronic disease that requires life-long gene expression.

### Are AAV vectors more suitable for CF gene therapy?

The other family of viruses developed for CF gene therapy are the adeno-associated viruses (AAVs), small nonenveloped, single-stranded DNA viruses, which also have a natural tropism for the lung and are nonpathogenic. The virus enters cells by binding to cell surface proteoglycans (sialic acid, galactose, or heparan sulfate)^60^ and to cell surface receptors such as fibroblast growth factor receptor or integrins.^61^ Although the wild-type virus integrates into the human genome, it has been suggested that the majority of AAV vectors remain in an episomal nuclear configuration after conversion from single-stranded into double-stranded DNA.^62,63^ However, Kaeppel and colleagues assessed the integration site profile in patients treated intramuscularly with alipogene tiparvovec (Glybera, an AAV vector carrying lipoprotein lipase) and showed that integration occurred but was random and, in contrast to gammaretroviral vectors, which have been associated with cases of insertional mutagenesis, not associated with preferential integration close to transcription start sites.^64^ Kaeppel and colleagues also noticed significant integration into mitochondrial genomes, which was surprising, and raises further questions related to AAV biology. One of the most attractive features of AAV vectors is their ability to support prolonged gene expression in many tissues.

The first AAV CF trial was published in 1998 and over a period of 8 years, six other clinical trials focused on delivering the vector by bronchoscope or by aerosol into nose, lung, or sinuses were conducted. These trials were led mainly by Targeted Genetics (now AmpliPhi Biosciences) and used serotype 2 (AAV2).^65^ Initial single-dose phase I trials demonstrated that virus administration to the CF airways was safe, but provided little opportunity to assess the efficiency of vector-specific CFTR expression. A large repeat-administration study (100 subjects), sufficiently powered to detect changes in lung function, did not meet its primary efficacy end point (improvement in lung function).^65^

There may be several reasons for these disappointing results: (1) AAV2 is too inefficient in transducing airway epithelial cells via the apical membrane; (2) the inverted terminal repeat (ITR) promoter, which was used to drive expression of the 4.7-kb *CFTR* cDNA because of the limited packaging capacity (about 5 kb) of the virus, had previously been shown to support CFTR expression when used *in vitro*,^66^ but is too weak *in vivo*; and/or (3) repeat administration of AAV2 to the lung is not possible because of the development of an antiviral immune response. No additional AAV lung trials have been performed since 2005, but research aimed at addressing and improving these potential limitations of AAV has been actively pursued (reviewed in Griesenbach and Alton^40^; and see below for discussion of some of the more recent studies) and some progress, particularly related to improving transduction efficiency, has been made.

However, on balance we would argue that AAV vectors may face similar problems with repeat administrations as adenoviral vectors, but a clinical trial assessing repeat administration of an AAV vector, proven to transduce the human airway epithelium efficiently, is the only way to address this question reliably. Interestingly, Liu and colleagues have shown that AAV vectors may be able to transduce progenitor cells in the mouse lung,^67^ which if efficient may help to overcome the repeat administration problem. In addition, Faust and colleagues have shown that depletion of CpG dinucleotides from the AAV vector reduces activation of Toll-like receptor 9 (TLR9)-mediated adaptive immune responses after intramuscular injection of the vector, leading to reduced inflammation and prolonged gene expression.^68^ However, it remains to be assessed whether CpG-depleted AAV vectors offer any advantages for CF gene therapy.

Several other AAV isoforms, either naturally occurring (e.g., AAV1, AAV5, and AAV6) or generated through rational design or directed evolution (e.g., AAV6.2), have been assessed in the lung and some have been shown to lead to higher transduction efficiency than the initial AAV2 vector.^69–73^ However, cross-species variation in vector transduction efficiency remains a challenging problem when deciding on the most appropriate *in vitro* or *in vivo* model system.^74^

As mentioned previously, the size of *CFTR* (∼4.7 kb) is close to the maximal packaging capacity of the virus,^75^ which leaves insufficient space for potent promoters and enhancers. Strategies based on using two AAV vectors as carriers for the transcription cassette (*trans*-splicing), which on cell entry recombine and generate a functional product, have been proposed. *Trans*-splicing-based AAV-CFTR transduction has led to partial correction of chloride transport *in vitro*,^70,76^ although significant questions related to the efficacy of this approach remain and translation into clinical trials has not yet been proposed. Yan and colleagues reported the generations of a chimeric virus consisting of a human bocavirus virus-1 (HBoV1) capsid, which comfortably accommodated an AAV2 genome carrying CFTR and patented transcriptional regulatory elements.^77^ The chimeric rAAV2/HBoV1 vector transduced polarized airway epithelial cells more efficiently than AAV1 and AAV2 *in vitro* and corrected CFTR-dependent chloride transport *in vitro*. It remains to be seen whether this vector configuration holds promise for *in vivo* applications.

Additional factors that may limit the efficacy of AAV vectors in CF lungs are the sputum and bacteria-induced inflammation. This is further supported by studies showing that CF sputum significantly restricts diffusion of AAV, which can in part by overcome by reducing AAV binding to the sputum and by decreasing sputum viscosity.^78^ In addition, Myint and colleagues used several bacteria-infection models to highlight that ongoing and recently resolved respiratory infections significantly decrease the efficacy of AAV-mediated gene transfer,^79^ which is relevant considering that the vast majority of patients with CF will suffer from pulmonary infections.

Although previously we have highlighted several limitations for using AAV vectors in the context of CF gene therapy, significant advances in vector development to overcome issues related to preexisting and adaptive immune responses and the limited packaging capacity have been made. Importantly, AAV vectors have proven clinical efficacy in the context of treating hemophilia^80^ and Leber's congenital amaurosis,^81^ and significant investments into the vector platform have been made by Pfizer^82^ and Sanofi-Genzyme.^83^

## Do RNA Viruses Hold Promise for CF Gene Therapy?

Paramyxoviruses such as human parainfluenza virus (hPIV) and murine Sendai virus (SeV) have been shown to transduce airway epithelial cells efficiently.^84^ Key factors for entry of both viruses into cells are the fusion (F) and hemagglutinin neuraminidase (HN) envelope proteins. The HN protein mediates virus–host cell attachment via sialic acid receptors, and the F protein is essential in fusing virus and cell membranes, allowing the viral nucleocapsid to be released into the host cell.^85^ hPIV achieved correction of the CF-related mucus transport defect *in vitro*,^27^ but only SeV was assessed *in vivo* and shown to partially correct CFTR-dependent chloride transport in CF knockout mice.^86^ However, SeV-mediated gene expression was transient (∼1 week) and the vector also induced strong adaptive immune responses that prevented efficient repeat administration of the vector.^87,88^

Lentiviral vectors have shown clinical efficacy in patients with Wiskott-Aldrich syndrome^89^ and metachromatic leukodystrophy.^90^ In addition, a trial in patients with Parkinson's disease demonstrated long-term safety and tolerability of direct injection of the vector into the brain.^91^ Lentiviruses are enveloped RNA viruses that belong to the family Retroviridae, and in contrast to gammaretroviruses, transduce dividing and nondividing cells,^92^ which make them a suitable vector for transduction of terminally differentiated cells in the airways.

Human (HIV),^93^ simian (SIV),^94^ and feline (FIV) immunodeficiency virus,^95^ as well as equine infectious anemia virus (EIAV) vector platforms,^96^ are being developed for CF gene therapy. These vectors do not have a natural tropism for the lung and therefore require pseudotyping with suitable envelope proteins to achieve efficient transduction. The vesicular stomatitis virus G (VSVG) protein, which is widely used, does not transduce airway epithelium efficiently via the apical membrane, but transduction efficiency can be increased by preadministration of tight junction openers that allow access of the virus to the basolateral membrane.^97^ However, it is currently unknown whether opening of tight junctions in the bacteria-infected CF lung is safe. Several other groups, including our own, have used lentiviral vectors pseudotyped with other envelope proteins such as influenza hemagglutinin (HA)^96^ and glycoprotein gp64 from baculovirus.^98^ We assessed an SIV vector pseudotyped with the F and HN proteins from SeV (rSIV.F/HN)^94^ and showed that the vector transduces airway epithelial cells efficiently and that a single dose achieved sustained gene expression for the lifetime of the mouse.^99,100^ It is currently unclear whether the sustained expression was due to a long half-life of airway epithelial cells and/or progenitor cell integration. Surprisingly, but consistent with data published by Sinn and colleagues,^95^ we also showed that the vector can be repeatedly administered without loss of efficacy,^99,100^ implying that lentiviral vectors are more effective in escaping adaptive immune response mechanisms than adenoviral and AAV vectors. These unique features make lentiviral vectors attractive candidates for further progression into CF gene therapy trials.

## Further Steps Taken to Progress Lentiviral Vectors Toward Clinical Trials in Patients with CF

1. Sinn and colleagues have shown that gp64-pseudotyped lentivirus transduced porcine airways *in vivo*, a first step toward assessing the efficacy of lentivirus-mediated gene transfer in CF knockout pigs.^101^
2. The U.K. Cystic Fibrosis Gene Therapy Consortium (CFGTC) has generated pharmacopoeia-compliant producer plasmids and developed scalable GMP-compliant production methods (CFGTC manuscript in preparation). We have conducted an extensive study to select the most appropriate and safest vector configuration for progression into clinical trial (CFGTC manuscript in preparation).
3. Insertional mutagenesis due to vector integration into the genomic DNA and possible activation of a proto-oncogene is a recognized safety concern for gammaretroviral vectors.^102^ It is important to note, however, that lentiviral vectors have not been associated with any serious adverse events resulting from insertional mutagenesis when used for bone marrow transduction in patients with metachromatic leukodystrophy and Wiskott-Aldrich syndrome.^89,90^ This appears to be due to improved vector design and differences in insertion site profiles between gammaretroviral and lentiviral vectors.^102^ However, in a β-thalassemia trial, lentiviral vector integration caused transcriptional activation of *HMGA2* in erythroid cells, leading to clonal dominance, which to date remains benign.^103^ Reports assessing the risk of insertional mutagenesis of lentiviral vectors in organs other than the bone marrow are limited. We, and others, have not seen any evidence of insertional mutagenesis after topical administration of lentiviral vectors to the intact airways,^95,100^ but extensive regulatory-compliant toxicology studies assessing acute and chronic toxicity as well as integration site profiling must be performed before progression to clinical trials.

The U.K. CFGTC is currently working toward a first-in-human phase I lentivirus CF gene therapy trial.

## Are Nonviral Gene Transfer Agents a Suitable Alternative to Viral Vectors for CF Gene Therapy?

Nonviral gene transfer formulations have two components: (1) the nucleic acid, that is, the therapeutic cDNA and appropriate regulatory elements; and (2) a carrier molecule that binds to the DNA. In the context of CF gene therapy a large number of carrier molecules have been developed that may be broadly characterized as either cationic lipids or cationic polymers. In preclinical airway *in vitro* and *in vivo* models nonviral vectors are generally less efficient than viral vectors,^40^ but a direct comparison in human lung has not yet been carried out.

The United Kingdom has been the center of activity for nonviral CF gene therapy. Caplen and colleagues conducted the first nonviral CF nose trial in 1995,^104^ and between 1995 and 2004 an additional six nonviral nose gene transfer studies (three in the United Kingdom) have taken place,^105–110^ all but one using cationic lipid formulations.^111^ In a further U.K. study in 2000, Hyde and colleagues established proof-of-concept that repeat administration (three doses) of a cationic liposome complexed to a plasmid carrying the human *CFTR* cDNA (pCFTR) to the nasal epithelium of patients with CF was feasible without loss of efficacy.^31^ Alton and colleagues, for the first time, administered the cationic lipid formulation GL67A complexed to pCFTR to the lungs of U.K. patients with CF and, encouragingly, electrophysiological changes could also be demonstrated in the lower airways. The study also showed that in contrast to the previous nose studies, administration of nonviral vectors to the lung caused mild transient inflammation.^30^ These finding were subsequently reproduced in the United States by Ruiz and colleagues.^108^ Combined, these studies showed that (a) gene transfer to the airway epithelium (nose and lung) was feasible, (b) partial correction of the CFTR-dependent chloride transport defect, but not the sodium transport defect, could be achieved in some, but not all, studies, and (c) administration of nonviral vectors was safe, but the nose did not predict the mild lung inflammatory responses.

As for all but one of the viral gene therapy trials, all nine nonviral gene therapy studies published to date were phase I/IIa safety studies, not designed to assess clinical benefit of gene therapy. The three U.K. centers involved in CF gene therapy trials based in London, Oxford, and Edinburgh formed the U.K. CFGTC in 2002 with the explicit aim to assess whether gene therapy can improve CF lung disease. From the start we anticipated that answering this question will require a long (∼12 months), double-blinded, placebo-controlled multidose trial sufficiently powered to assess clinical benefit. Since 2002 the U.K. CFGTC has conducted a phased translational research program (Table 2) and key stages are described below.

**Table 2.** U.K. Cystic Fibrosis Gene Therapy Program

*Phase*	*Objectives*	*Progression criteria*
Preclinical selection and development of a GTA suitable for repeated administration to patients with CF	Selection of pGM169/GL67A	1. Transfection of AECs 2. Low toxicity 3. Repeat administration feasible 4. Stability in nebulizer 5. GMP production feasible
Tracking study	Validation of putative end-point assays in patients undergoing exacerbations	Selection of end points that respond to conventional treatment
Single-dose phase I/IIa pilot trial	1. Selection of suitable dose for MDT 2. Confirmation of dosing interval in patients with CF	1. Suitable dose: 5 ml of pGM169/GL67A per dose 2. Dosing interval: Monthly for 12 months
Run-in study	1. Selection of primary and secondary end points 2. Characterization of most suitable patient population for MDT trial	1. Primary end point: Percent change in relative FEV_1_ from baseline 2. Inclusion of patients >12 years and with baseline FEV_1_ of 50 to 90%
Multidose murine and ovine regulatory-compliant toxicology studies	Prove safety in animal models	1. No chronic inflammation 2. No remodeling of lung 3. No extrapulmonary effects
Multidose, double-blinded, placebo-controlled phase IIb trial	Significant difference in primary end point comparing active and placebo groups	Depending on outcome of trial

AECs, airway epithelial cells; CF, cystic fibrosis; FEV_1_, forced expiratory volume in the first second; GMP, Good Manufacturing Process; GTA, gene transfer agent; MDT, multidose trial.

1. Activity in the 1990s led to the development of a myriad of nonviral gene transfer agents, but it is our impression that progress in developing novel formulations for airway gene therapy has been modest and that GL67A, which was used in the first U.K. nonviral lung trial (see previously) in 1999 remains the most potent nonviral vector for airway gene transfer more than a decade later.^112^ We also compared a range of commercially available nebulizers to select the most appropriate device for aerosol delivery of pGM169/GL67A complexes.^113^
2. The mild inflammatory responses and transient duration of gene expression noted by Alton and colleagues after single-dose administration of GL67A/pCFTR aerosols to the lungs of patients with CF^30^ led to the development of CpG-nucleotide free plasmids containing a synthetic transcriptional regulator consisting of the elongation factor-1α promoter and the human cytomegalovirus (CMV) enhancer.^114^ This novel CpG-free plasmid led to reduced inflammation by avoiding activation of Toll-like receptor (TLR)-9 pathways, and prolonged (>4 weeks) gene expression in mouse models.
3. A study aimed at selection of putative end-point assays based on their responses to conventional antibiotic treatments during an exacerbation (tracking study; see Table 2) allowed the identification of key biomarkers of inflammation, imaging, and physiology that alter alongside symptomatic improvement after treatment of an acute CF exacerbation. These data, in parallel with our study of biomarkers in patients with stable CF (run-in study; see Table 2), provided important guidance in choosing optimal biomarkers for subsequent gene therapy trials.^115^
4. After selection of the most potent nonviral gene transfer agent (pGM169/GL67A) a single-dose phase I/IIa open-label safety trial was conducted to select the most appropriate dose of pGM169/GL67A for the subsequent multidose safety trial. This trial included assessment of molecular efficacy (correction of CFTR-dependent chloride transport in lungs and nose of treated patients) (CFGTC manuscript in preparation).
5. A study aimed at (a) selection of putative end-point assays based on their sensitivity and variability in stable patients and at (b) selection of the most appropriate patient population (run-in study; see Table 2) included approximately 200 patients. During trial visits, which were scheduled approximately every 6 months, patients underwent a range of assays including a one-off deposition scan. A key factor for patient selection is to find a good balance between “can deliver” (not too severe to prevent GL67A/pDNA deposition) and “can measure” (not too well to mask clinical efficacy).
6. Before progression into a multidose clinical trial, regulatory-compliant toxicology studies were performed. Mice received 12 doses of pGM169/GL67A over 6 months and sheep received 9 doses of pGM169/GL67 at monthly intervals.^116,117^ All animals tolerated the treatment well. We did not observe any chronic inflammatory responses, lung remodeling, or any extrapulmonary responses and the no-observed adverse event limit (NOAEL) supported progression into a multidose clinical trial.
7. The multidose, double-blinded placebo controlled phase IIb trial designed to assess clinical efficacy started in summer 2012 and was completed 2 years later. The trial included patients 12 years and older with an FEV_1_ between ≥50% to ≤90% predicted. All mutation types were included, although, as expected, the vast majority of patients were F508del homo- or compound heterozygotes. Patients received 12 monthly 5-ml doses of pGM169/GL67A containing 13.25 mg of plasmid DNA complexed to 75 mg of GL67A (or placebo [saline]) by aerosol. The primary end point was a relative change in percent predicted FEV_1_. Secondary outcomes included additional measurements of lung function, CT scans, and Cystic Fibrosis Questionnaire-revised (CFQ-R). Exploratory end points included exercise testing, activity monitoring, and sputum inflammatory markers. Mechanistic end points included nasal and/or bronchial vector-specific DNA, mRNA, and electrophysiological assessment of CFTR function. Safety was extensively evaluated. Results from 116 patients (54 placebo, 62 active) are being analyzed and, depending on the outcome, may be either further assessed in phase III studies or may require further improvement in efficacy and consistency of response.

## Conclusions

Research into CFTR correctors and, more importantly, the potentiator Kalydeco, has shown that the molecular defect of CFTR is drug targetable and that amelioration of CFTR function translates into improvements in disease phenotype. These findings have renewed interest and investment into other therapies aimed at targeting the molecular defect, including gene therapy. In contrast to the mutation class–specific treatments described previously, gene therapy is mutation class independent and will likely be suitable for all patients. However, proof-of-concept that gene therapy can improve CF lung disease is currently lacking and the results of the U.K. CF Gene Therapy Consortium's nonviral phase IIb trial, which has been completed, are awaited. Lentiviral vectors hold promise for CF gene therapy because of their long-lasting expression and efficacy on repeated administration.

Although not covered in this review, it is also important to briefly consider genome editing in the context of CF. Strategies based on zinc finger nucleases, transcription activator-like effector nucleases (TALENs), and more recently CRISPR–Cas9 are widely used to generate *in vitro* and *in vivo* models.^118^ In the context of CF, Schwank and colleagues used the CRISPR–Cas9 technology to correct the *CFTR* locus in human and mouse intestinal organoids.^119^ Similarly, Lee and colleagues used zinc finger nucleases to correct the F508del mutation in an *in vitro* model.^120^ However, genome editing of the *CFTR* locus is currently more suited to the development of preclinical models, rather than of therapeutic value in the context of CF lung therapy.
